# How Are FOMO and Nomophobia Linked to Symptoms of Depression, Anxiety and Stress Among University Students?

**DOI:** 10.1192/bjo.2024.104

**Published:** 2024-08-01

**Authors:** Hamid Alhaj, Abdelraouf Muthana, Asia Abdalla, Menna Marouf, Nisreen Awad

**Affiliations:** University of Sharjah, Sharjah, UAE

## Abstract

**Aims:**

Nomophobia, defined as the fear of being without one's mobile phone, and FOMO (Fear of Missing Out) are on the rise and are thought to be linked to increased mental health problems. In the information era, being separated from smartphones may cause anxiety, while the expectation of continuous updates on social media may increase feelings of inadequacy and distress when comparing one's life with selected highlights of others. The extent of nomophobia and FOMO in the Middle East and whether these experiences are associated with psychiatric disorders are yet to be ascertained. The purpose of this study was to determine the prevalence of nomophobia and FOMO among university students in the UAE and the relationship between these phenomena and depression, anxiety and stress levels.

**Methods:**

232 female and 103 male undergraduate students in four Emirates (Abu Dhabi, Dubai, Sharjah, and Ajman) took part in the study. An online questionnaire was developed and piloted. Nomophobia and FOMO were measured using validated questionnaires, namely NMP-Q and FoMOs. Symptoms of depression, anxiety and stress were assessed using the DASS-21 scale. Data were analysed using SPSS 22. Significance level was set at p < 0.05.

**Results:**

The data revealed that 28.6% of respondents exhibited severe, 47.7% moderate, and 23.7% mild nomophobia symptoms. 52.5% of participants reported moderate to extreme fear that others have more rewarding experiences than them, with the median FoMO score being (25.62). Higher nomophobia, stress, anxiety, and depression levels correlated with elevated FOMO scores (p < 0.001). Variations in FOMO scores were noted across university, gender, and college. Strong associations existed between severe nomophobia and heightened stress, anxiety, and depression (p < 0.001). The findings underscored contextual influences on nomophobia intensity among diverse individuals.

**Conclusion:**

The study identified a high prevalence of nomophobia and FOMO among UAE university students. Significant correlations were observed between these digital-related fears and mental health issues like depression, anxiety, and stress. Our results delineate the necessity for exploring and implementing interventions that address smartphone-related phobias to safeguard the mental well-being of UAE university students, considering their unique cultural context.

